# Genomic evidence reveals high genetic diversity in a narrowly distributed species and natural hybridization risk with a widespread species in the genus *Geodorum*

**DOI:** 10.1186/s12870-023-04285-w

**Published:** 2023-06-14

**Authors:** Xianliang Zhu, Jianmin Tang, Haidu Jiang, Yishan Yang, Zongyou Chen, Rong Zou, Aizhu Xu, Yajin Luo, Zhenhai Deng, Xiao Wei, Shengfeng Chai

**Affiliations:** 1 Guangxi Key Laboratory of Plant Functional Phytochemicals and Sustainable Utilization, Guangxi Institute of Botany, Guangxi Zhuang Autonomous Region and Chinese Academy of Sciences, Guilin, 541006 China; 2 College of Tourism and Landscape Architecture, Guilin University of Technology, Guilin, Guangxi 541006 China; 3 Yachang Orchid National Nature Reserve Management Center, Baise, Guangxi 533209 China

**Keywords:** Conservation genomics, *Geodorum eulophioides*, *G. densiflorum*, Gene flow, Orchidaceae, SNP

## Abstract

**Background:**

Understanding genetic diversity is a core issue in conservation genetics. However, previous genetic diversity evaluations of narrowly distributed species have rarely used closely related widespread species as a reference. Furthermore, identifying natural hybridization signals between narrowly and widely distributed sympatric species is of great importance for the development of species conservation programs.

**Methods:**

In this study, population genotyping by sequencing (GBS) was performed for a narrowly distributed species, *Geodorum eulophioides* (endemic and endangered in Southwest China), and a widespread species, *G. densiflorum*. A total of 18,490 high-quality single nucleotide polymorphisms (SNPs) were identified at the whole-genome level.

**Results:**

The results showed that the nucleotide diversity and heterozygosity of *G. eulophioides* were significantly higher than those of *G. densiflorum*, confirming that narrowly distributed species can still preserve high genetic diversity. Consistent with taxonomic boundaries, all sampled individuals from the two species were divided into two genetic clusters and showed high genetic differentiation between species. However, in a sympatric population, a few *G. eulophioides* individuals were detected with genetic components from *G. densiflorum*, suggesting potential interspecific natural hybridization. This hypothesis was supported by Treemix analysis and hand-hybridization trials. Invasion of the habitat of *G. eulophioides* invasion by *G. densiflorum* under anthropogenic disturbance may be the main factor causing interspecific hybridization.

**Conclusions:**

Therefore, reducing or avoiding habitat disturbance is a key measure to protect the *G. eulophioides* populations. This study provides valuable information for future conservation programs for narrowly distributed species.

**Supplementary Information:**

The online version contains supplementary material available at 10.1186/s12870-023-04285-w.

## Background

Genetic diversity is an important component of biodiversity and is the sum of all genetic information carried by different individuals within a species or a population [1]. Understanding the degree of genetic diversity in narrowly distributed species is a core issue in conservation genetics [2]. Traditionally, most narrowly distributed species have been thought to have lower genetic diversity than widespread species [3, 4], although this view has been challenged [5]. In addition, the genetic diversity of species is influenced by factors other than distribution range, including evolutionary history, longevity, mating systems, and seed dispersal mechanisms [6]. This complexity makes it difficult to compare the degree of genetic diversity between widely and narrowly distributed species under the same criteria. This challenge has been previously resolved in some related species with similar life history traits and reproductive mechanisms [7–10]. Here, we used two related species belonging to the genus *Geodorum*: a narrowly distributed species and a widespread species.

**Fig. 1:**
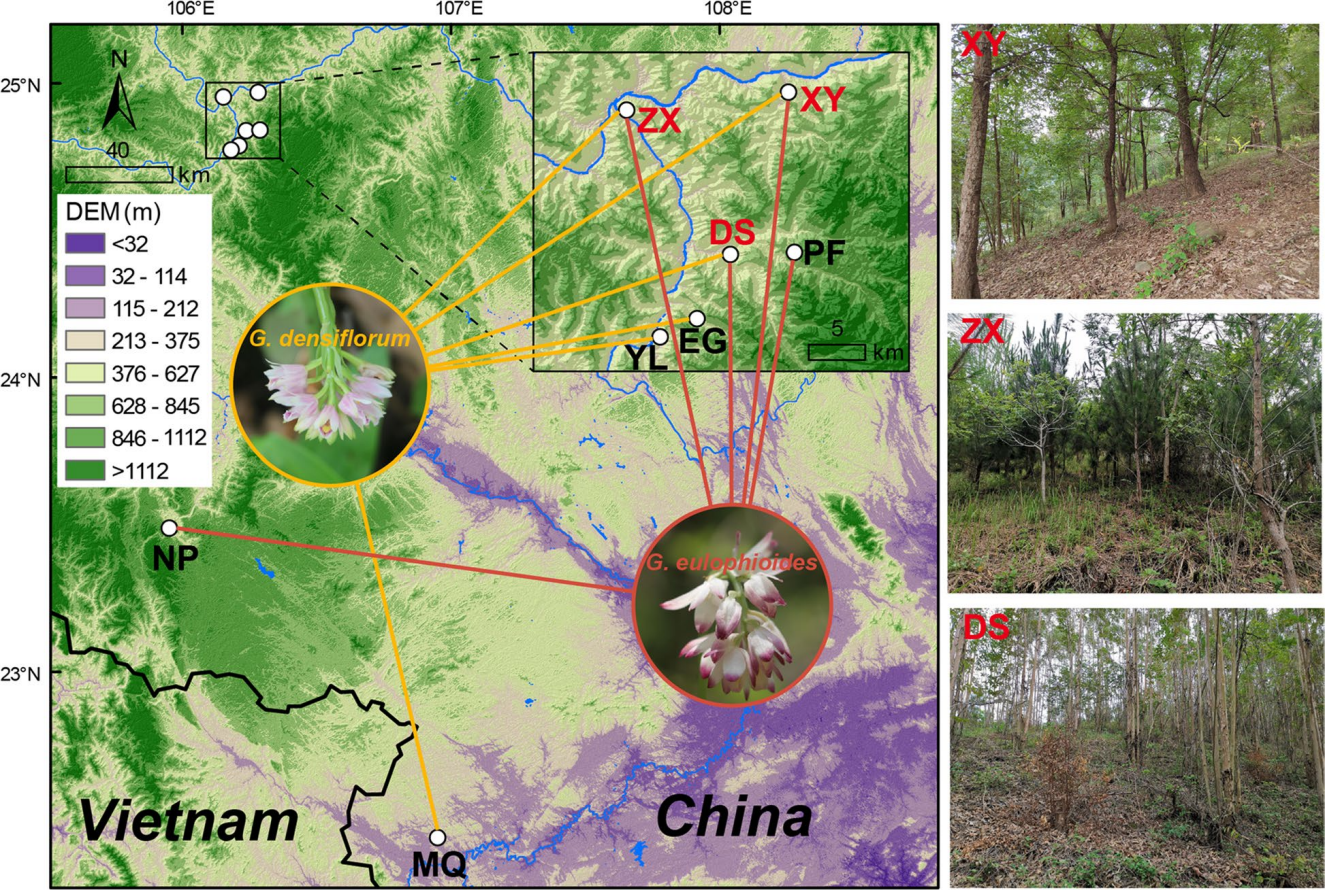
Sampling locations of *G. eulophioides* and *G. densiflorum*. The map was downloaded from Geospatial Data Cloud (https://www.gscloud.cn). DEM refers to the digital elevation mode. The habitats of the sympatric populations (XY, ZX, and DS) of the two species are shown in the images on the right

The genus *Geodorum* is a ground-growing type of herb in the Orchidaceae family with medicinal and ornamental values, comprising approximately ten species [11]. *Geodorum eulophioides* is an orchid endemic to Southwest China. The species was first discovered and named by a German plant taxonomist Schlechter in 1921 in Luodian, Guizhou province. However, this species has not been found in the wild for more than 80 years. In 2004, *G. eulophioides* was rediscovered in Yachang, Guangxi Zhuang Autonomous Region. Due to the extremely narrow range of this species in the wild, *G. eulophioides* has been classified as endangered on the Threatened Species List of China’s Higher Plants [12]. At present, *G. eulophioides* is only narrowly distributed in parts of Guangxi, Guizhou, and Yunnan provinces in China. This species prefers shade and grows primarily in canyons, broad-leaved forests, and thickets at about 600 m elevation. The number of extant plants of *G. eulophioides* is very low. The total number of individual plants found in the Yachang Orchid Nature Reserve in Guangxi does not exceed 400, while several field populations with a small population distribution of 104 plants in total have been found in Guizhou [11, 13]. *Geodorum densiflorum* is a typical widespread species with distribution in Guangxi, Guangdong, Taiwan, Hainan, and other provinces in China, as well as India, Vietnam, Malaysia, Japan, and other countries in Asia. It is sun-loving and grows primarily in sparse forests, streamsides, and grassy slopes below 1500 m elevation. The flower structure and color of *G. densiflorum* differ markedly from those of *G. eulophioides* (Fig. 1). However, these two species share a close flowering period (mid-June to early July) and common insect-type pollinators [14]. Furthermore, the two species occur in a sympatric distribution in northern Guangxi under anthropogenic habitat disturbance. These factors may provide opportunities for natural hybridization between the species [15]. Although a previous study demonstrated a low incidence of natural hybridization between *G. densiflorum* and *G. eulophioides* through comparative reproductive biology [14], this result is, however, yet to be supported by genomic evidence.

Natural hybridization between species may contribute to increasing a population’s genetic diversity, promoting environmental adaptability, and potentially creating new species [16]. Based on genomic evidence, Fu et al. [17] recently demonstrated that two sympatric *Quercus* species (*Q. acutissima* and *Q. variabilis*) improved population fitness via hybridization. Nevertheless, natural hybridization between widespread and narrowly distributed species may result in some negative effects. For example, hybridization may cause genetic and demographic swamping and accelerate the extinction of narrowly distributed species [18]. Zhou et al. [19] identified natural hybridization between a narrowly distributed species (*Melastoma penicillatum*) and a widespread species (*M. candidum*), based on a few low-copy nuclear genes and chloroplast DNA intergenic spacers. They hypothesized that the result may be detrimental to the conservation of the narrowly distributed species. In fact, a precedent for natural hybridization has been found in orchids [20]. Therefore, identifying natural hybridization signals between *G. densiflorum* and *G. eulophioides* species through genomic evidence is of great important for the development of conservation programs.

Single nucleotide polymorphisms (SNPs) are the most abundant variant form in the genome and are highly stable and easily detected [21]. SNPs are increasingly used for species conservation, accelerating the shift from conservation genetics to conservation genomics [22]. However, for species with large genome sizes, the cost of obtaining genome-wide SNPs remains a challenge. The published genomes of several orchid species are large in size and complex in structure [23, 24]. Thus, an economical and efficient SNP-genotyping strategy may be more suitable for orchids, such as genotyping by sequencing (GBS) [25]. In previous studies, GBS has been shown to be superior in assessing genetic diversity, genetic structure, and interspecific gene flow [26–28]. In the genus *Geodorum*, a previous study used SNPs generated by double digest restriction site-associated DNA sequencing (RAD-seq) data to assess the genetic diversity of *G. densiflorum* [29]. In contrast, only a few simple sequence repeat (SSR) markers were used by Ying [30] to assess genetic diversity and population structure in *G. eulophioides*. To date, genome-wide SNP data have not been previously used to assess the genetic diversity and population structure of *G. eulophioides*.

The main objectives of this study were to use genome-wide SNP data (1) to compare the levels of genetic diversity between the narrowly distributed *G. eulophioides* and the widespread *G. densiflorum*, (2) to investigate the genetic differentiation and population structure between the two species, and (3) to detect the possibility of natural hybridization between the two species, and to provide meaningful conservation strategies and suggestions for narrowly distributed species.

## Results

### Genetic diversity

After filtering, a total of about 334 G clean bases were obtained in 38 *G. eulophioides* individuals from five populations and 52 *G. densiflorum* individuals from six populations, as well as three *Eulophia graminea* individuals, using GBS (Fig. 1 and Table S1). The average Q20 and Q30 for each sample were 97.26% and 93.54%, respectively (Table S1). The average GC content of *G. eulophioides* (46.76%) was somewhat higher than that of *G. densiflorum* (45.89%). A total of 4,911,676 raw SNPs were called using Stacks’s de novo pipeline. After linkage disequilibrium (LD) pruning and filtering, 18,490 high-quality SNPs were obtained for further downstream analysis.

**Fig. 2:**
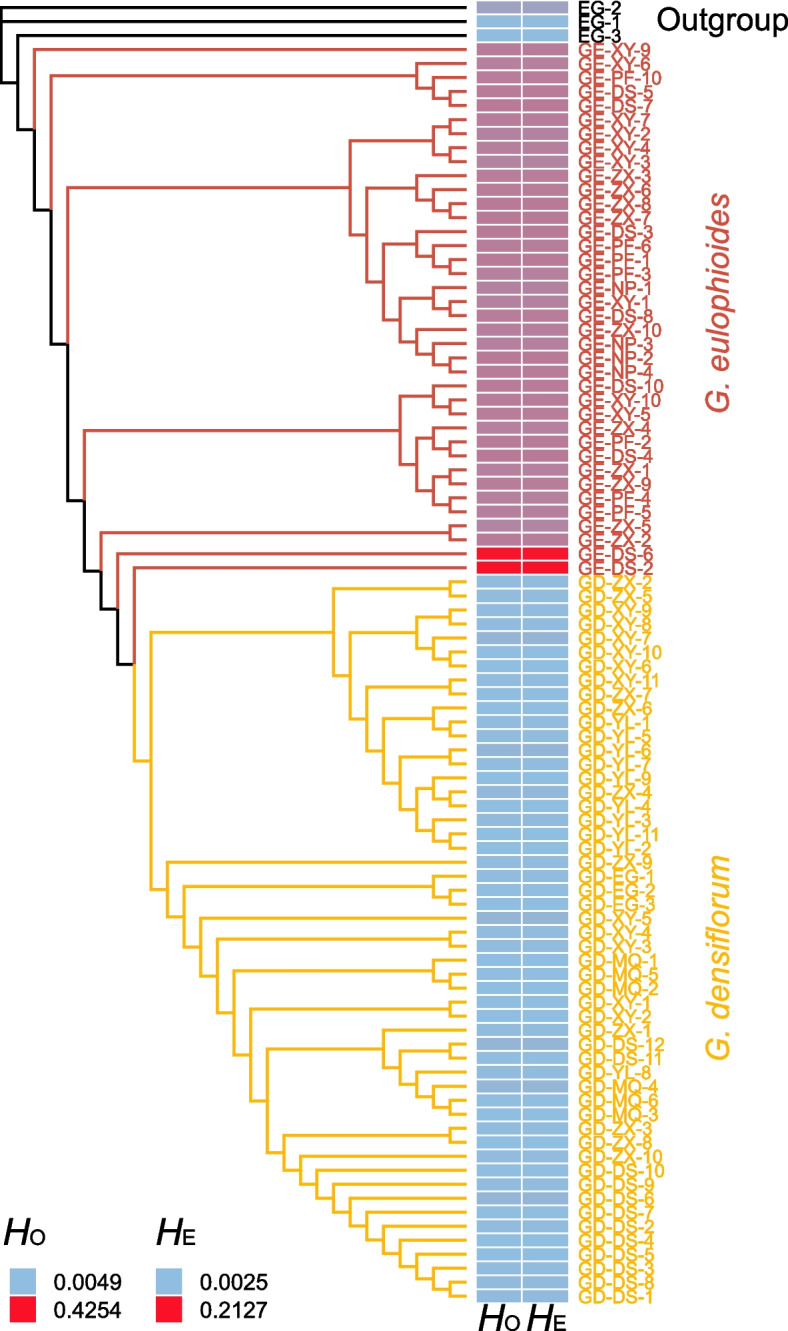
Maximum likelihood tree and heterozygosity values at the individual level. The heatmap outside the branches shows the individual *H*_E_ and *H*_O_ values

Using 18,490 SNPs, we compared the genetic diversity level between *G. eulophioides* and *G. densiflorum.* At the species level, the expected heterozygosity (*H*_E_) (0.0247) and observed heterozygosity (*H*_O_) (0.0117) of *G. densiflorum* were both significantly lower than that of *G. eulophioides* (*H*_E_, 0.1553; *H*_O_, 0.1822) (Table 1). The nucleotide diversity (π) value of *G. eulophioides* (0.1580) was about six times higher than that of *G. densiflorum* (0.0250). These results may be related to the higher number of private alleles in *G. eulophioides*. Similar results were observed at the population level, with the highest π in *G. eulophioides* in the GE-DS population (0.2063) and *G. densiflorum* in the GD-ZX population (0.0262). At the individual level, we found that the heterozygosity of two individuals (GE-DS-6, and GE-DS-2) was significantly higher than that of other individuals (Fig. 2). In addition, all *G. eulophioides* populations showed negative inbreeding coefficients (*F*_IS_) while, in comparison, only one *G. densiflorum* population showed a negative *F*_IS_ value. In addition, Tajima’s D values were positive for all populations except for the GE-DS population (Table 1).

**Table 1:** Genetic diversity at the species and population levels

Species/Population	*n*	*N*p	*H*_O_	*H*_E_	π	*F*_IS_	Tajima’D
*G. eulophioides*	38	15,015	.1822(.0017)	.1553(.0012)	.1580(.0012)	-.0351(.0280)	-
GE-DS	8	766	.2257(.0018)	.1902(.0012)	.2063(.0013)	-.0377(.0093)	-.0594
GE-NP	4	198	.1728(.0023)	.1132(.0014)	.1395(.0018)	-.0582(.0062)	.5717
GE-PF	7	390	.1706(.0021)	.1274(.0013)	.1435(.0015)	-.0519(.009)	.4207
GE-XY	9	547	.1698(.0019)	.1295(.0013)	.1403(.0014)	-.0606(.0108)	.2598
GE-ZX	10	582	.1718(.0019)	.1319(.0013)	.1416(.0014)	-.0624(.0114)	.2713
*G. densiflorum*	52	857	.0117(.0007)	.0247(.0007)	.0250(.0007)	.0471(.0170)	-
GD-DS	12	15	.0099(.0007)	.0104(.0004)	.0110(.0005)	.0149(.0094)	.1039
GD-EG	3	0	.0099(.0007)	.0052(.0004)	.0070(.0005)	-.0047(.0026)	-
GD-MQ	6	5	.0109(.0006)	.0134(.0006)	.0148(.0006)	.0075(.0045)	1.1961
GD-XY	11	4	.0139(.0007)	.0217(.0007)	.0228(.0007)	.0255(.0046)	.6233
GD-YL	10	8	.0121(.0007)	.0227(.0007)	.0239(.0007)	.0328(.0048)	.6433
GD-ZX	10	16	.0113(.0007)	.0248(.0007)	.0262(.0008)	.0414(.0048)	.5884

*n* number of individuals,* N*p Number of private alleles, *H*_O_ observed heterozygosity, *H*_E_ expected heterozygosity, π nucleotide diversity, *F*_IS_ inbreeding coefficient. The values in parentheses represent the standard error

### Genetic structure

**Fig. 3:**
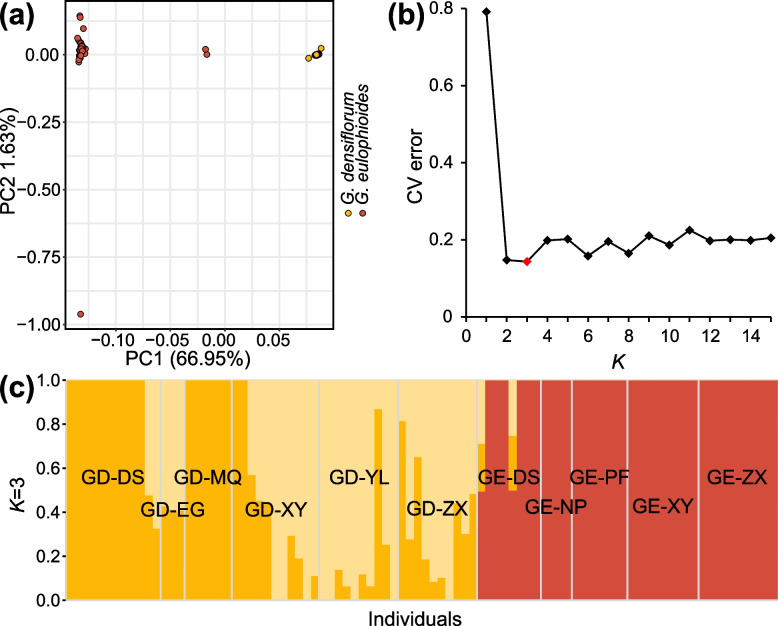
Genetic structures of *G. eulophioides* and *G. densiflorum*. **a** Principal component analysis (PCA) plot generated for the genome-wide SNP data. **b** Cross-validation (CV) errors for *K* = 1–15. **c** Genetic structure bar plots at *K* = 3

The maximum likelihood (ML) phylogenetic tree of the individuals showed that *G. eulophioides* and *G. densiflorum* were divided into two different clades, while GE-DS-6 and GE-DS-2 in *G. eulophioides* were the two individuals most closely related to *G. densiflorum* (Fig. 2). The principal component analysis (PCA) was consistent with the ML tree and individuals of *G. densiflorum* and *G. eulophioides* were clearly distinguishable, with PC1 and PC2 explaining 66.95% and 1.63% of the variation, respectively (Fig. 3a). The cross-validation (CV) error was minimized when *K* = 3 (Fig. 3b). In this scenario, Admixture analysis showed that there was a clear genetic structure between *G. densiflorum* and *G. eulophioides*, suggesting that the two species contained different ancestral components (Fig. 3c). Meanwhile, *G. densiflorum* showed a more complex origin than *G. eulophioides*. However, an ancestral component from *G. densiflorum* was observed in two individuals (GE-DS-6 and GE-DS-2) in the GE-DS population of *G. eulophioides*, suggesting the possible presence of interspecific gene flow.

### Genetic differentiation and gene flow

**Fig. 4:**
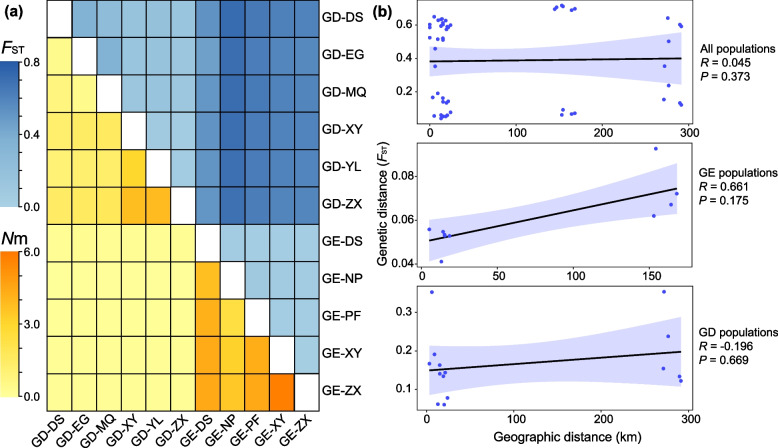
Genetic differentiation (*F*_ST_), gene flow (*N*m), and Mantel test. The top right of **a** indicates *F*_ST_, and the bottom left indicates *N*m between populations. **b** is the Mantel test for *F*_ST_ and geographic distances for all 11 populations and five *G. eulophioides* (GE) populations, six *G. densiflorum* (GD) populations. *R* and *P* represent the Spearman’s correlation coefficient and significance, respectively

**Fig. 5:**
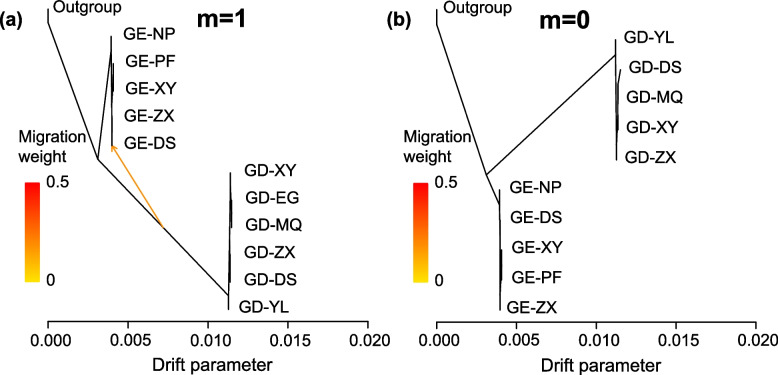
Gene flow analysis using Treemix. **a** and **b** are the maximum likelihood trees of the best migration model (m) detected using all individuals and after the removal of admixed individuals. The outgroup is *Eulophia graminea*

The mean genetic differentiation coefficient (*F*_ST_) (0.061) between the *G. eulophioides* populations was lower than that of *G. densiflorum* (0.263) (Fig. 4a). Correspondingly, the mean gene flow (*N*m) within the populations of *G. eulophioides* and *G. densiflorum* were 4.064 and 1.695, respectively (Fig. 4a). However, genetic differentiation between populations of the two species was very high (*F*_ST_ between 0.459 and 0.717) (Fig. 4a). The potential effect of geographic distance on genetic differentiation was analyzed using the Mantel test and the results showed that there was no significant correlation between both global and local *F*_ST_ and geographic distance (Fig. 4b). To further assess interspecific gene flow, ML trees and variance interpretation for 0–5 migration events were simulated in Treemix. The optimal migration pattern (m = 1) was determined from the residuals under each model, and gene flow from the *G. densiflorum* to the DE-DS population was detected (Fig. 5a and Fig. S1). To find evidence of older genetic introgression, all admixed individuals identified in the genetic structural analysis were removed. Treemix was then rerun for the remaining 65 individuals and three outgroups. In this reanalysis, the optimal migration model was m = 0, suggesting that there was no ancient introgression between *G. eulophioides* and *G. densiflorum* (Fig. 5b and Fig. S2).

### Hybridization of *G. eulophioides* and *G. densiflorum*

The results of the hand-hybridization trial showed that fruit was obtained successfully either with *G. eulophioides* as the female and *G. densiflorum* as the male, or with *G. densiflorum* as the female and *G. eulophioides* as the male (Table 2), confirming that the two species could be hybridized.

**Table 2:** Fruit set determined by hand-hybridization between *G. eulophioides* and *G. densiflorum*

Trials	No. of marked or hybridized flowers	No. of fruits	Fruit set (%)
*G. eulophioides* controls	61	6	9.84
*G. densiflorum* controls	82	15	18.29
*G. eulophioides* × *G. densiflorum*	11	6	54.55
*G. densiflorum* × *G. eulophioides*	12	8	66.67

## Discussion

### Genetic diversity levels of the narrowly distributed *G. eulophioides* and the widespread *G. densiflorum*

For biallelic SNP markers, nucleotide diversity (π) is an overall indicator of population genetic diversity, with higher π values indicating higher genetic diversity [31, 32]. The estimated π value (0.0250) for *G. densiflorum* was close to that previously reported by Roy et al. [29]. The results of the Roy et al. [29] study were based on the ddRAD-seq for *G. densiflorum* (0.03586) distributed in India and between three other orchids (*Dendrobium densiflorum*, 0.10560; *Rhynchostylis retusa*, 0.11344; *Cymbidium aloifolium*, 0.01364), suggesting that the genetic diversity of *G. densiflorum* may generally be at a low to moderate level. In *G. eulophioides*, Ying [30] used 18 SSR markers to calculate the *H*_E_ and *H*_O_ values of 0.6968 and 0.7797, respectively. These values were higher than in this study based on SNP data (*H*_E_, 0.1553; *H*_O_, 0.1822). Due to the differences in the estimation of genetic diversity using different molecular markers, it was difficult to make substantial comparisons across studies. However, many previous studies have shown that the use of only a few SSR markers may overestimate the heterozygosity of the population, compared to the use of large-scale genome-wide SNP loci [33, 34]. Using large-scale SNPs generated by GBS, we confirmed that the genetic diversity level of the narrowly distributed species, *G. eulophioides*, was significantly higher than that of the widespread species, *G. densiflorum*.

The genetic diversity of widespread and narrowly distributed species may be influenced by many factors, such as evolutionary history, longevity, mating systems, and seed dispersal mechanisms [6]. We comprehensively analyzed the potential impact of these factors on the genetic diversity of the two species. On the one hand, Tajima’s D values showed that most of the *G. eulophioides* and *G. densiflorum* populations were positive (Table 1), suggesting that the two species may have similar evolutionary histories of population shrinkage or equilibrium selection. It was interesting to note that the Tajima’s D values observed for the *G. densiflorum* populations were opposite to those previously found in India [29], suggesting that *G. densiflorum* may have a different evolutionary history in different regions. On the other hand, as two closely related species, *G. eulophioides* and *G. densiflorum* exhibited similarities in many life-history traits, such as longevity, seed dispersal mechanisms, and the presence of ground-growing varieties, but may also have different mating patterns [11]. By studying comparative reproductive biology, Lin [14] found that the selfing rate of *G. densiflorum* was much higher than the outcrossing rate, while the selfing rate of *G. eulophioides* was comparable to the outcrossing rate. Compared to other *Geodorum* species, the poor ability of *G. eulophioides* to self-fertilize is a key contributor to this species becoming endangered [11]. This result was also supported by the *F*_IS_ values, where evidence of outcrossing (*F*_IS_ < 0) was detected in all *G. eulophioides* populations (Table 1). However, most *G. densiflorum* populations had *F*_IS_ values greater than 0, indicating that selfing or inbreeding was universal in *G. densiflorum*. Selfing is considered to be an effective strategy to increase plant population size, as it guarantees the reproduction of the species when pollinators are limited [35, 36]. Thus, *G. densiflorum* with selfing as the main mating system can maintain a higher population size and wider distribution range, but at the cost of possibly lower levels of genetic diversity. On the contrary, outcrossing of *G. eulophioides* can lead to the continuous accumulation of genetic variation and thus the acquisition of greater genetic diversity. Furthermore, it was found in the hybridization trials that the fruit set of *G. eulophioides* was lower than that of *G. densiflorum* in the wild (Table 2). In summary, we suggest that the mating system may be an important reason for the considerable differences in genetic diversity between *G. eulophioides* and *G. densiflorum*. Further, however, a true understanding of the effects of selfing or outcrossing on species genetic diversity requires further sampling from ripened ovaries of donor plants that have been selfed or out-crossed within and among populations versus leaf samples.

### Genetic differentiation and gene flow between *G. eulophioides* and *G. densiflorum*

Orchids are generally insect-pollinated [37], as is the case with the genus *Geodorum*. Lin [14] observed that *Ceratinidia cognata* was a shared pollinator of both in the sympatric distribution of *G. densiflorum* and *G. eulophioides*. However, the frequency of the pollinator visiting *G. eulophioides* was higher than that of *G. densiflorum*. Thus, it could explain the lower *F*_ST_ and higher *N*m we observed among *G. eulophioides* populations compared to *G. densiflorum* (Fig. 4a). In addition, considering that *G. densiflorum* reproduced more often via selfing, the gene flow between their populations may be weaker than that of *G. eulophioides*.

Consistent with taxonomic boundaries, all sampled individuals of the two species were divided into two genetic clusters and showed high genetic differentiation between species (Figs. 2, 3 and 4). However, no significant isolation by distance (IBD) signal was found in either the global or local Mantel test (Fig. 4b), implying that geographic isolation was not a major factor contributing to the high degree of interspecific differentiation between *G. densiflorum* and *G. eulophioides*. One explanation for this phenomenon may be due to their habitat specificity. *G. eulophioides* is particularly shade-loving and forest depths with moderate shade are one of the preferred habitats of this species. *G. densiflorum* is also habitat-specific, such as the forest edges or sparse forests with more sunlight. Thus, even at close geographic distances, two species may be segregated due to local habitat differences, thereby weakening the IBD pattern. Moreover, in habitats with severe anthropogenic disturbances, similar patterns of IBD deficiency have been found in some narrowly distributed or endangered plants in China, such as *Brasenia schreberi* [38], *Cryptomeria japonica* [39], and *Tetraena mongolica* [40]. As such, the high genetic differentiation and IBD deficiency among species in the genus *Geodorum* may also have been subject to anthropogenic pressures.

Some previous studies have shown that gene flow may also exist between species with high genetic differentiation, due to ancient incomplete lineage sorting or recent hybridization [41–43]. Based on the Admixture results (Fig. 3c), the two *G. eulophioides* individuals (GE-DS-6 and DE-DS-2) in the DS population were more likely to be the result of interspecific hybridization [41]. This hypothesis was supported by Treemix, where a significant interspecies gene flow from the *G. densiflorum* to the GE-DS population was observed. The Admixture results could also be due to other factors, such as ancient gene flows [44]. However, such ancient gene flows were not detected in the Treemix analysis after the exclusion of admixed individuals (Fig. 5). The hand-hybridization trials confirmed the possibility of hybridization between the two species (Table 2). Combined with the planting history of the DS population, it was found that a large number of eucalyptus trees had been introduced by residents long before the establishment of the Yachang Orchid Nature Reserve. Although the eucalyptus was removed in 2008 and 2009, sprouted eucalyptus trees remain the dominant species in the DS population (Fig. 1). It is important to note that the canopy density of eucalyptus plantations is usually very low [45], providing more suitable habitat for *G. densiflorum*. If the habitat preference of *G. densiflorum* and *G. eulophioides* is followed, they rarely have the opportunity to have overlapping habitats. Anthropogenic habitat disturbance may be an important factor for the occurrence of actual sympatric distribution of the two orchid species. We hypothesized that during the initial natural forest-dominated period of the DS population, which was a more suitable habitat for *G. eulophioides*, and during the subsequent plantation period, *G. densiflorum* gradually invaded the *G. eulophioides* habitat, and during their sympatric period, natural hybridization between the two species may have occurred, resulting in interspecific gene flow. However, in the other two sympatric populations (ZX and XY), no evidence of interspecific gene flow was detected (Fig. 5). This may be due to the fact that insect-mediated interspecific hybridization may be highly accidental and conditions such as temperature, light, and humidity in different habitats may affect the success rate of interspecific hybridization [46, 47]. Although the ZX and XY populations also have a history of plantation forestry, they are almost all native species (mainly mason pine and tung tree, see Fig. 1) with long felling cycles and thus may have received less anthropogenic disturbance. Alternatively, species hybridization between other sympatric populations may have occurred, but at very low levels, and too few individuals were investigated to be able to detect this accurately. Overall, this study confirms that natural hybridization between the two orchid species is possible. Further genomic studies of these potential natural hybrids and true artificial hybrids, or population genetic analyses combined with plastid genomic data, could better explore hybridization among species of the genus *Geodorum*.

### Mechanisms of endangerment and conservation recommendations for *G. eulophioides*

It is generally accepted that the causes of species endangerment include two main aspects. One part is the species itself (e.g. low level of genetic diversity) and the other is external ecological factors (e.g. habitat destruction and environmental changes) [48, 49]. Both these issues should be fully considered when developing species conservation plans. *G. eulophioides* maintains high genetic diversity, suggesting that ecological factors may be the main cause of their endangerment. The destruction and loss of habitat suitable for plants are detrimental to population size and thus further affect life history. Most narrowly distributed species are poorly adapted to the environment and have difficulty responding effectively when the environment changes [50, 51]. Alternatively, widespread species often possess greater habitat tolerance and adaptive plasticity, withstanding environmental disturbances and avoiding the risk of extinction, compared to the narrowly distributed species [52]. There are some benefits to natural hybridization, for example, in terms of heterozygosity, the two *G. eulophioides* individuals (GE-DS-6 and GE-DS-2, see Fig. 2) were more than the other individuals, which implies they may have higher adaptive capacity. However, it is uncertain whether the more adaptable hybrid progeny will threaten the survival of the already endangered narrowly distributed species. In addition, considering the natural hybridization of the narrowly distributed *G. eulophioides* and the widespread *G. densiflorum* may occur under anthropogenic habitat disturbances. Reducing or avoiding habitat disturbance may be key to the conservation of narrowly distributed species [19]. With the establishment of the Yachang Orchid Nature Reserve, the habitat protection of *G. eulophioides* has been greatly improved and their population size has also been somewhat enhanced. However, according to our field survey, the canopy density of *G. eulophioides* habitat in the reserve was not very high. Therefore, creating a more suitable habitat for *G. eulophioides* may assist their populations to grow and avoid *G. densiflorum* invasion. For example, increasing the canopy of the dominant tree species through pruning techniques or planting additional broad-leaved trees to provide a better shade environment.

## Conclusions

In this study, the genetic variation and differentiation between the narrowly distributed species *G. eulophioides* and the widespread species *G. densiflorum* were investigated using 18,490 SNP markers. The results showed that the genetic diversity of *G. eulophioides* was higher and this species showed significant genetic differentiation from *G. densiflorum*. However, interspecific gene flow was detected in a sympatric population (DS) from *G. densiflorum* to *G. eulophioides*, which may natural hybridization signal. Invasion of the *G. eulophioides* habitat by *G. densiflorum* under anthropogenic disturbance may be the main factor causing interspecific hybridization. Therefore, reducing or avoiding habitat disturbance is a key measure to protect the *G. eulophioides* populations. This study provides valuable information for future conservation programs for the endangered orchid.

## Methods

### Plant materials

Leaf samples were collected from 38 *G. eulophioides* individuals from five populations and 52 *G. densiflorum* individuals from six populations (Fig. 1 and Table S1). Among them, both *G. eulophioides* and *G. densiflorum* were distributed in DS, XY, and ZX populations, which were termed sympatric populations. The NP and PF populations contained only *G. eulophioides*, and the YL, EG, and MQ populations contained only *G. densiflorum*. The DS, EG, PF, XY, YL, and ZX populations were located in Yachang Orchid Nature Reserve of Guangxi and its surrounding areas. In addition, we collected leaf samples of three *Eulophia graminea* individuals, which were from the ZX population. Due to the small size of some populations, a larger sampling distance means fewer representative samples within the population, so we balanced the total number of samples with the sampling spacing to obtain as many representative samples as possible. All sampled plants were spaced more than 10 m apart and their fresh leaves were dried and preserved in allochroic silica gel. Dr. Shengfeng Chai from Guangxi Institute of Botany conducted the plant material collection and the formal identification for this study. One voucher specimen was collected for each population and deposited in the herbarium of the Guangxi Institute of Botany (code 20210623001–20220702001, see Table S1). The collection of leaves and voucher specimens of *G. eulophioides* in this study was permitted by the Yachang Orchid National Nature Reserve Management Center, and the collecting of all materials complied with the Regulations of the People’s Republic of China on the Protection of Wild Plants and the IUCN Policy Statement on Research Involving Species at Risk of Extinction.

### Genotyping by sequencing

Genomic DNA of 93 samples was extracted using the E.Z.N.A. Tissue DNA kit (Omega Bio-Tek, USA). The quantified DNA (> 3 μg; concentration > 30 ng/μl) was used for ddGBS library construction using the *EcoRI*/*NlaIII* enzyme combination, according to the protocol of Biozeron Biotechnology Co., Ltd (Shanghai, China). The final pooled libraries were sequenced on an Illumina Novaseq 6000 platform with a paired-end 150-bp read length. Quality assessment of reads was performed with FastQC [53]. Adapters and low-quality reads (Q20 < 20, length < 36 bp) of the raw data were filtered using Trimmomatic v0.36 [54]. We used the de novo pipeline of Stacks v2.59 [55], including the *ustacks*, *cstacks*, *sstacks*, *tsv2bam*, and *gstacks* modules, for SNP calling. Next, LD pruning of the raw SNP dataset was performed using *populations* module of Stacks with the parameter “-write-random-snp”. The LD-pruned SNPs were then filtered using VCFtools v0.1.16 [56] using the following criteria: max missing rate of 0.3, min quality of 20, min and max allelic of 2, min mean depth of 3, and minor allele frequency of 0.05.

### Genetic diversity

The *populations* module of Stacks was used to calculate the π, *H*_O_, *H*_E_, and *F*_IS_. The Tajima’s D values of each population were estimated using VCFtools, with a sliding window size of 3000 bp. Since the calculation of Tajima’s D value requires at least four individuals, the GD-EG population was excluded.

### Genetic structure

A phylogenetic tree was constructed using the ML method in IQtree v2.0 [57], using the “-m MFP” parameter to test all models and select best-fit model (GTR + F + R3) according to the Akaike information criterion and the “-bb 1000 -bnni -alrt 1000” parameter to calculate the node support. *E. graminea* individuals were used as outgroups. The phylogenetic tree was visualized using the *Phylogenetic tree view* function of ImageGP [58]. The Plink v1.07 [59] was used to perform PCA and calculate the eigenvectors of each principal component. Bayesian clustering was utilized for all individuals using Admixture v1.3.0 [60] and the assumed number of substructures (*K*) were set from 1 to 15. The optimal *K* value was determined based on the minimum CV error.

### Genetic differentiation and gene flow

The *F*_ST_ between populations was calculated using VCFtools. Based on the geographic coordinate information of the population, the actual geographic distance was calculated using the R package *geosphere*. The Mantel test was then performed using the R package *vegan* for *F*_ST_ and geographical distance with Spearman’s correlation coefficient and 9999 permutations to test significance. The interpopulation *N*m was calculated according to the equation: ${\text{Nm}}{=}{(1-\text{F}_{\text{ST}})}/{4}{\text{F}}_{\text{ST}}$. Concurrently, migration events between populations were analyzed using Treemix v1.12 [61]. The assumed number of migration events (m) was set between 0 and 5. Using the “-root” parameter to set *E. graminea* as an outgroup, and the “-noss -k 500” parameters were added to prevent over-correction of sample sizes, and reconstruct the ML tree by resampling blocks of 500 SNPs. Residuals were used to choose the best-fit migration model. According to the Treemix recommendation [61], when the constructed model is able to explain 99.8% of the data, the addition of migration edges can be stopped, at which point the model can already adequately explain the current gene flow situation.

### Hand-hybridization trial

To verify the possibility of hybridization between *G. eulophioides* and *G. densiflorum*, we randomly selected five *G. eulophioides* and five *G. densiflorum* individuals in the wild to conduct hand-hybridization trials. Flowers that were about to bloom were sequentially emasculated, bagged, and pollinated according to the method described by Dafni [62]. Eight wild *G. eulophioides* and ten *G. densiflorum* individuals were marked, and their fruit set under natural conditions was recorded as controls. The fruit-set rate was recorded three months after the trial was conducted. Since an individual may produce more than one flower, the fruit set was counted by the number of flowers.

## Supplementary Information

**Additional file 1: Fig. S1.** The corresponding residuals when using all individuals to simulate 0–5 migration events (m). SE, standard errors.**Fig. S2.** The corresponding residuals when using individuals after removing admixtures to simulate 0–5 migration events (m). SE, standard errors. **Table S1.** Sampling and sequencing information.

## Data Availability

Raw GBS data are available at NCBI with the SRA accession number of PRJNA888812.

## Abbreviations

CV: Cross-validation
*F*_IS_: Inbreeding coefficient
*F*_ST_: Differentiation coefficient
GBS: Genotyping by sequencing
*H*_E_: Expected heterozygosity
*H*_O_: Observed heterozygosity
IBD: Isolation by distance.
LD: Linkage disequilibrium
ML: Maximum likelihood
*N*m: Gene flow
PCA: Principal component analysis
RAD-seq: Restriction site-associated DNA sequencing
SNP: Single nucleotide polymorphisms
SSR: Simple sequence repeat
π: Nucleotide diversity
