# Supplementary material for: Genomic evidence reveals high genetic diversity in a narrowly distributed species and natural hybridization risk with a widespread species in the genus Geodorum
Source: BMC Plant Biol. 2023 Jun 14;23:317. doi: 10.1186/s12870-023-04285-w (PMC10265804; doi:10.1186/s12870-023-04285-w)
Supplement: Supplementary file 1 — Additional file 1: Fig. S1. The corresponding residuals when using all individuals to simulate 0–5 migration events (m). SE, standard errors.Fig. S2. The corresponding residuals when using individuals after removing admixtures to simulate 0–5 migration events (m). SE, standard errors. Table S1. Sampling and sequencing information. [file 12870_2023_4285_MOESM1_ESM.zip › Supplementary file_ESM.docx]

## Supplementary file


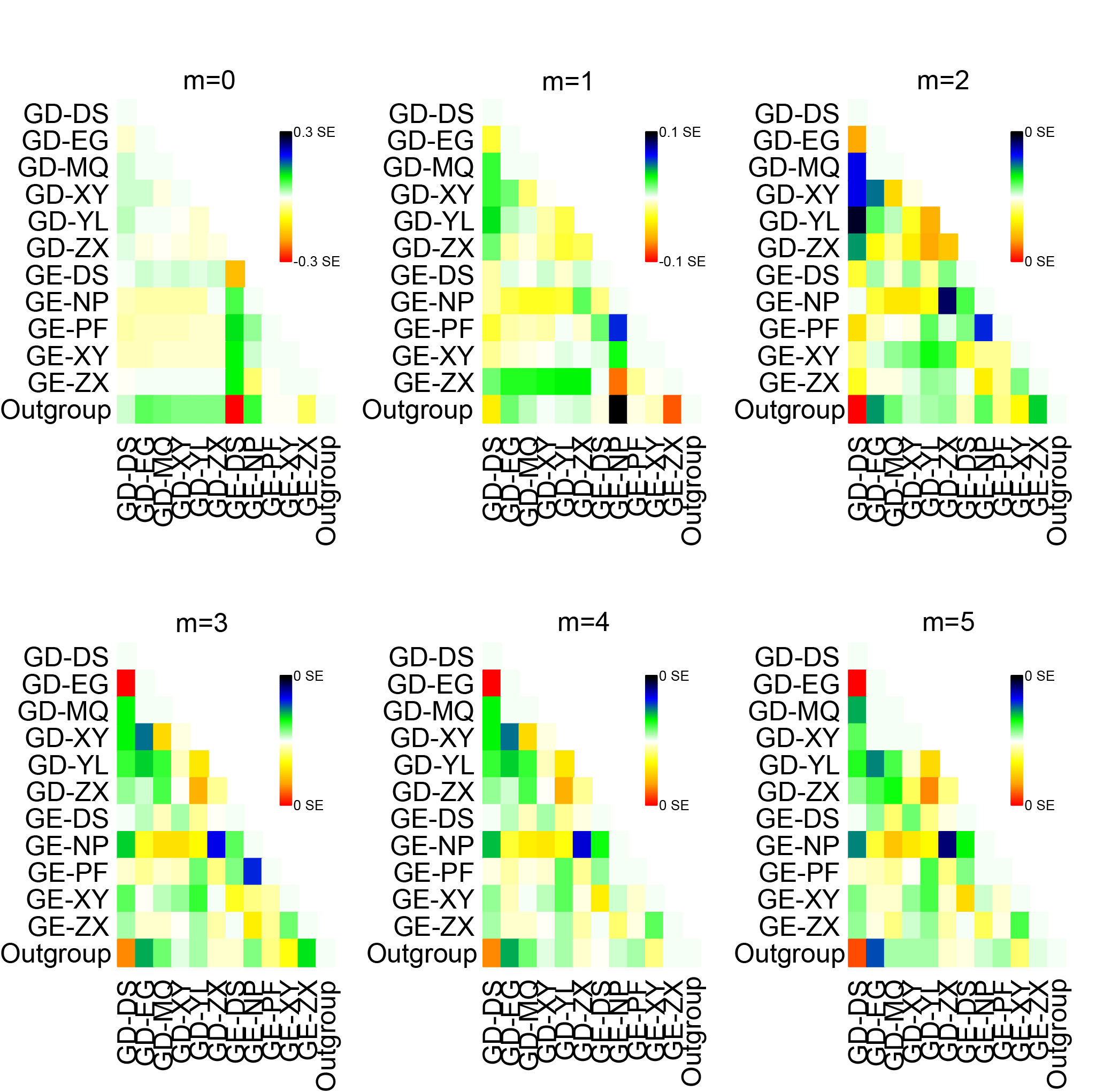


**Fig. S1 The corresponding residuals when using all individuals to simulate 0–5 migration events (m).** SE, standard errors.


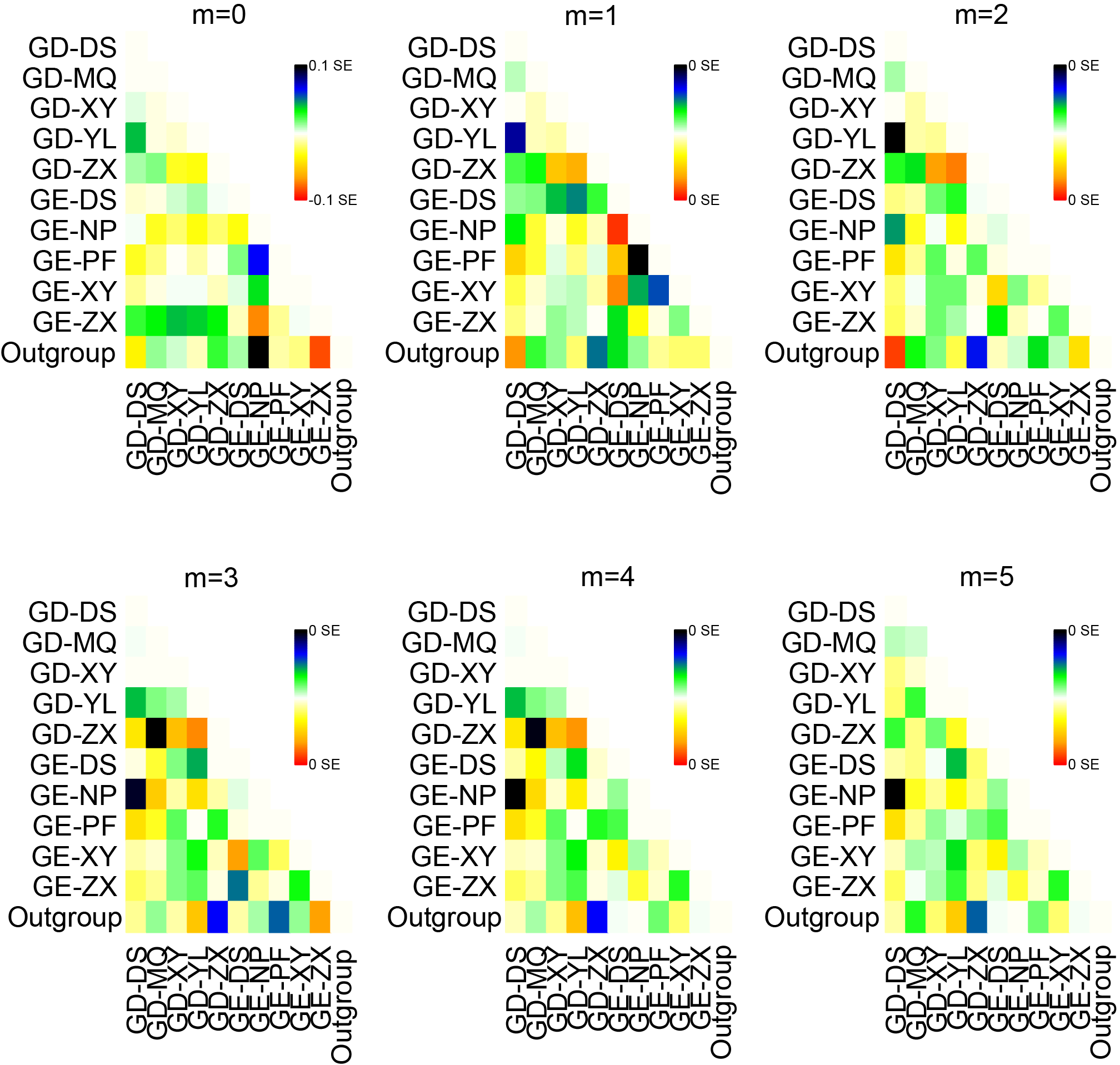


**Fig. S2 The corresponding residuals when using individuals after removing admixtures to simulate 0–5 migration events (m).** SE, standard errors.

**Table S1 Sampling and sequencing information.**
